# Involvement of APOBEC3B in mutation induction by irradiation

**DOI:** 10.1093/jrr/rraa069

**Published:** 2020-09-03

**Authors:** Yohei Saito, Hiromasa Miura, Nozomi Takahashi, Yoshikazu Kuwahara, Yumi Yamamoto, Manabu Fukumoto, Fumihiko Yamamoto

**Affiliations:** Department of Radiopharmacy, Tohoku Medical and Pharmaceutical University, Sendai, Japan; Department of Radiopharmacy, Tohoku Medical and Pharmaceutical University, Sendai, Japan; Department of Radiopharmacy, Tohoku Medical and Pharmaceutical University, Sendai, Japan; Department of Radiation Biology and Medicine, Faculty of Medicine, Tohoku Medical and Pharmaceutical University, Sendai, Japan; Department of Radiopharmacy, Tohoku Medical and Pharmaceutical University, Sendai, Japan; Department of Pathology, Institute of Development, Aging and Cancer, Tohoku University, Sendai, Japan; Department of Molecular Pathology, Tokyo Medical University, Tokyo, Japan; Department of Radiopharmacy, Tohoku Medical and Pharmaceutical University, Sendai, Japan

**Keywords:** DNA repair, radiation effects, mutation rate, APOBEC3

## Abstract

To better understand the cancer risk posed by radiation and the development of radiation therapy resistant cancer cells, we investigated the involvement of the cancer risk factor, APOBEC3B, in the generation of radiation-induced mutations. Expression of *APOBEC3B* in response to irradiation was determined in three human cancer cell lines by real-time quantitative PCR. Using the hypoxanthine-guanine phosphoribosyl transferase (*HPRT*) mutation assay, mutations in the *HPRT* gene caused by irradiation were compared between APOBEC3B-deficient human hepatocellular carcinoma (HepG2) cells [APOBEC3B knocked out (KO) using CRISPR-Cas9 genome editing] and the parent cell line. Then, *HPRT*-mutated cells were individually cultured to perform PCR and DNA sequencing of *HPRT* exons. X-Irradiation induced APOBEC3B expression in HepG2, human cervical cancer epithelial carcinoma (HeLa) and human oral squamous cell carcinoma (SAS) cells. Forced expression of APOBEC3B increased spontaneous mutations. By contrast, APOBEC3B KO not only decreased the spontaneous mutation rate, but also strongly suppressed the increase in mutation frequency after irradiation in the parent cell line. Although forced expression of APOBEC3B in the nucleus caused DNA damage, higher levels of APOBEC3B tended to reduce APOBEC3B-induced γ-H2AX foci formation (a measure of DNA damage repair). Further, the number of γ-H2AX foci in cells stably expressing APOBEC3B was not much higher than that in controls before and after irradiation, suggesting that a DNA repair pathway may be activated. This study demonstrates that irradiation induces sustained expression of APOBEC3B in HepG2, HeLa and SAS cells, and that APOBEC3B enhances radiation-induced partial deletions.

## INTRODUCTION

Radiation-induced cancer risk depends on the increased DNA damage and the mutations that occur during the repair process. Radiation-induced chromosomal aberrations and gene mutations have been studied extensively. It is established that, although radiation causes all classes of mutations, radiation-induced mutations are commonly indels [1, 2]. Although the DNA repair mechanism leading to indels, non-homologous end joining (NHEJ), is increasingly well-understood, radiation-induced carcinogenesis is thought to also require other gene mutations, caused by additional factors, the details of which are unknown.

Irradiation (IR) changes the activation of transcription factors involved in the cell cycle and apoptosis, which control the expression levels of many genes, processes collectively considered as the radiation response [3, 4]. Abnormal radiation response function leads to an increase in incomplete DNA repair and mutations. Our preliminary experiments revealed that in HepG2–8960-R, which is a radioresistant cell line derived from human hepatocarcinoma cell line HepG2 cells by repeated radiation exposure [5], the cytidine deaminase, APOBEC3B, is expressed at higher levels than in the parent line (Supplementary Fig. 1, see online supplementary material). The APOBEC3 family consists of seven members with polynucleotide cytidine deaminase activity, and mainly acts on single-stranded DNA (ssDNA) of retroviruses and retrotransposons [6]. According to a recent comprehensive genome sequence analysis, a characteristic kataegis mutation pattern is prevalent in breast cancer, the generation of which is likely to involve APOBEC3B [7]. High expression of APOBEC3 in cancer cells is correlated with an increased frequency of genome-wide GC to AT mutations [8]. Further, APOBEC3B can mutate the lagging strand during DNA replication [9], and enforced expression of APOBEC3A and APOBEC3B has been reported to cause DNA damage, resulting in reduced cell viability [10]. Although there is poor correlation between APOBEC3B expression levels in normal tissues and cancer risk, increased APOBEC3B expression is predicted to promote mutations in cancer cells. *In vitro*, APOBEC3B can cause mutations of foreign DNA, such as transfected plasmids [11]. Further, APOBEC3B is postulated to be responsible for tumor heterogeneity and may contribute to the acquisition of therapeutic resistance [12, 13].

A relationship between APOBEC3G and radiosensitivity has been demonstrated in several studies [14, 15]; however, it is not entirely clear whether other APOBEC3 family members influence radiosensitivity or radiation-induced mutations. We hypothesized that APOBEC3B accelerates radiation-induced mutagenesis, and examined the involvement of APOBEC3B in the radiation-induced increases in gene mutations, using APOBEC3B knockout (KO) cells. The present study reveals that APOBEC3B, a known oncogene, plays a role in radiation-induced mutation in cancers.

## MATERIALS AND METHODS

### Cell culture and genome editing

HepG2, human cervical cancer epithelial carcinoma (HeLa) and human oral squamous cell carcinoma (SAS) cell lines were obtained from the Cell Resource Center for Biomedical Research, Institute of Development, Aging and Cancer, Tohoku University. The clinically relevant radioresistant (CRR) HepG2–8960-R cell line was established from HepG2 [5]. Cells were cultured in RPMI1640 (Nacalai Tesque, Inc., Kyoto, Japan) containing 5% fetal calf serum (FCS; Biological Industries, Kibbutz Beit Haemek, Israel) in a humidified 5% CO_2_ atmosphere at 37°C. APOBEC3B KO cells were established from HepG2 using a CRISPR-Cas9 genome-editing system (Dharmacon, Lafayette, CO, USA). The cells have a deletion across the intron 2/exon 3 border of the APOBEC3B gene. Plasmids are described in the online supplementary material.

### Irradiation

X-Ray irradiation (1 Gy/min) was performed using a 150 KVp X-ray generator (Meditec Japan Corporation, Chiba, Japan) with a filter.

### Western blotting

Cells were plated into 12-well plates and incubated overnight. After exposure to IR, the cells were lysed in SDS sample buffer and separated by SDS-PAGE. Proteins were transferred to Immobilon-P membrane (Millipore, Billerica, MA, USA), and were then blocked and incubated with each anti-APOBEC3 antibody ( see online supplementary material) or anti-actin polyclonal antibody (Sigma-Aldrich, St. Louis, MO, USA). Proteins were detected using Luminata Crescendo Western HRP Substrate (Millipore) and medical X-ray Film Super RX (FUJIFILM Corporation, Tokyo, Japan).

### Real-time PCR

cDNAs were generated using an RNeasy kit (QIAGEN GmbH, Hilden, Germany) and the SuperScript III First-Strand Synthesis System for RT-PCR (Thermo Fisher Scientific). *APOBEC3* gene family expression levels were determined using SYBR GREEN I qPCR assays (THUNDERBIRD SYBR qPCR Mix, TOYOBO Co., Ltd.) and the StepOnePlus Real-Time PCR System (Thermo Fisher Scientific). Primer sequences are provided in Supplementary Table 1, see online supplementary material. Real-time quantitative PCR (qPCR) data were analyzed by the comparative CT method, using beta-actin as the reference standard.

### HPRT assay and determination of survival fraction

To calculate the spontaneous mutation rate, the HPRT assay using HepG2 cells was designed with reference to Glaab and Tindall [16]. *HPRT*-mutant cells were eliminated by culturing for 1 month in medium supplemented with hypoxanthine, aminopterin and thymidine (HAT; Thermo Fisher Scientific) prior to initiating the HPRT assay. Mutant frequency was determined initially and at several time points while maintaining the cells in logarithmic growth. To perform 6-thioguanine (6-TG) selection, HAT-cleansed cells were then incubated for 1 day without HAT and plated in the presence of 6-TG at a density of 10^6^ cells per 10 cm dish; 5 × 10^2^ cells per 10 cm dish were plated in triplicate without HAT to obtain a plating efficiency (PE) at the time of selection. In addition, 2 × 10^6^ cells were subcultured without HAT for a subsequent 6-TG selection. Additional 6-TG selection and subculture were performed every 3 days. The total cell number in the subculture was obtained prior to each 6-TG selection, using PE from the previous selection. After 2–3 weeks, 6-TG-resistant colonies and colonies on PE plates were stained with 0.5% crystal violet and counted. Mutation frequency was calculated according to the following equation: mutation frequency = (total number of 6-TG-resistant colonies/10^6^) × (total number of colonies on three PE plates/1.5 × 10^3^). Population doubling was calculated according to the following equation: population doubling = (ln [total number of cells]−ln [number cells plated × PE])/ln2. Mutation rate (mutations/cell/generation) was then obtained by plotting the observed mutant frequency as a function of population doubling and calculating the slope by linear regression analysis using Graphpad Prism 8 (MDF Co., Tokyo, Japan).

To determine the IR-induced mutation frequency and survival fraction following IR, HAT-treated cells were cultured for 1 week in growth medium without HAT to allow expression of mutant phenotypes. Cells were then cultured in a 10 cm dish with 6-TG for 3 weeks to allow colony formation. 6-TG-resistant colonies were individually cultured in 12-well plates for *HPRT* exon PCR and DNA sequencing. To determine the plating efficiency and the fraction of cells surviving, 500 or 2000 cells were plated in a 10 cm dish without 6-TG. Cells were cultured for 1–2 weeks, and then colonies were stained with 0.5% crystal violet and counted.

### HPRT exon PCR and DNA sequencing

Genomic DNA was extracted from *HPRT*-mutant cells using a Wizard Genomic DNA purification kit (Promega, Madison, WI, USA). Each exon deletion was detected using PrimeSTAR MAX DNA polymerase and two different PCR primer sets (Supplementary Table 2, see online supplementary material) using the PCR conditions described in the online supplementary material. PCR amplicons were electrophoresed on a 1.5% agarose gel, and gel images were captured using an ATTO Printgraph. Bands of interest were cut out of the gel, and the DNA was extracted and sequenced (FASMAC, Kanagawa, Japan). Sequencing primers sequences are presented in Supplementary Table 2.

### High-content image analysis

Cells (5 × 10^3^/well) were plated into 96-well plates (Cell Carrier Ultra-96; PerkinElmer, Waltham, MA, USA) and cultured overnight. After IR with 5 Gy, cells were fixed with 4% paraformaldehyde for 20 min, permeabilized, and then stained with Alexa Fluor 488-conjugated anti-γ-H2AX Ab and Hoechst 33342. Cell images were analyzed using an Operetta CLS High-Content System (PerkinElmer).

For three-color imaging, cells expressing a GFP-APOBEC3B fusion protein were stained with an anti-γ-H2AX antibody, followed by a secondary antibody conjugated to Alexa Fluor 594, and Hoechst 33342.

### Statistical analysis

Survival fraction, mutation frequency and high-content image analysis data were analyzed using Student’s *t*-test. Mutation rates were analyzed using regression analysis. Results are expressed as the mean ± SEM of triplicate measurements unless otherwise stated. The ratios of radiation-induced mutations to spontaneous mutations were analyzed using a Chi-square test with Yates’ correction. Confidence intervals (CIs) (95%) were calculated.

## RESULTS

### IR induces APOBEC3B expression

**Fig. 1.**
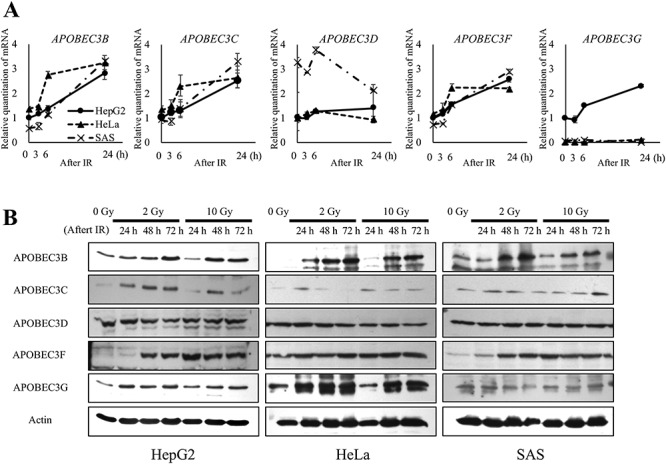
mRNA and protein expression of the APOBEC3 family in response to irradiation. (**A**) After a 10 Gy X-ray dose, the cells were incubated for 3, 6 or 24 h, and then total RNA was extracted. Real-time RT-PCR was performed using primers to amplify *APOBEC3B*, *APOBEC3C, APOBEC3D, APOBEC3F* and *APOBEC3G*. The mRNA expression levels at each time point were calculated relative to those of unirradiated HepG2 cells. (**B**) Cells were irradiated with 2 or 10 Gy X-ray (1 Gy/min); incubated for 0, 24, 48 or 72 h; and then lysed to perform immunoblotting for APOBEC3B, APOBEC3C, APOBEC3D, APOBEC3F, APOBEC3G and actin.

The CRR cell line, HepG2–8960-R, was treated with 2 Gy IR every day to maintain its traits [5]. HepG2–8960-R had a higher level of APOBEC3B protein expression (Supplementary Fig. 1, see online supplementary material) and a higher mutation frequency than the parent HepG2 cell line [17]. Since it may not only be genomic instability but also the high level of APOBEC3B expression that contributes to the high mutation rate of HepG2–8960-R, we first examined whether radiation stress induced the expression of APOBEC3 family members (except 3A and 3H) in the HepG2, HeLa and SAS cell lines, which are all parent lines of established CRR cells. Exposure to 10 Gy IR increased the mRNA expression of *APOBEC3B*, *APOBEC3C* and *APOBEC3F* ~2-fold or more in all cells after 6 and 24 h, as determined by qPCR (Fig. 1A); however, *APOBEC3G* mRNA was only elevated in HepG2 cells (PCR amplification products were not confirmed in HeLa or SAS). Further, APOBEC3B protein expression was upregulated between 24 and 72 h after IR in all cells (Fig. 1B). To investigate whether the elevated expression of APOBEC3B after IR caused an increase in mutation frequency, we knocked out APOBEC3B in HepG2 cells (A3BKO); APOBEC3B KO was confirmed by PCR and western blotting (Supplementary Fig. 2A and B, see online supplementary material). The expression level of APOBEC3 family members, other than APOBEC3B, in A3BKO cells, HepG2 cells expressing APOBEC3B-tGFP (A3B-tGFP) and A3BKO cells expressing APOBEC3B-tGFP (A3BKO + A3B-tGFP) was similar to that in the parent HepG2 cell line (Supplementary Fig. 2C). All subsequent experiments were performed using the HepG2 cell line and its derivatives.

### KO of APOBEC3B suppresses spontaneous and IR-induced mutations

**Fig. 2.**
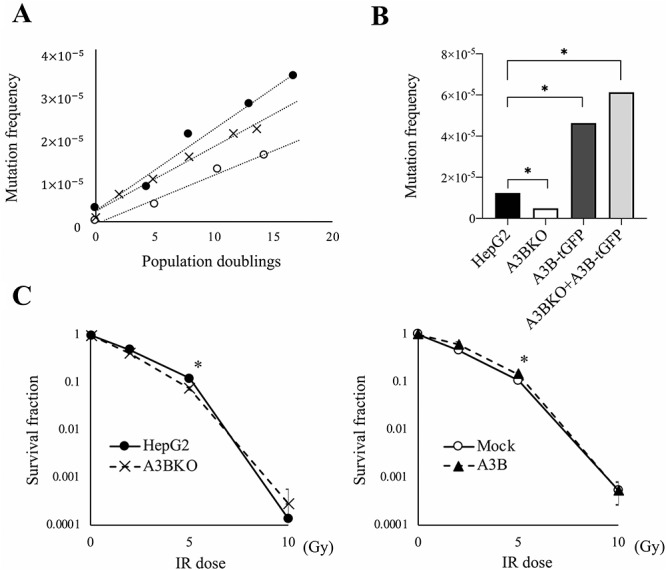
Knocking out APOBEC3B decreases the spontaneous mutation rate. (**A**) The mutation rates of HepG2 (filled circle), A3BKO (empty circle) and control Cas9-expressing cells (cross) were determined by plotting mutation frequencies vs population doublings and calculating the slope by linear regression. The slope of the curve yields the mutation rate (mutations/cell/generation). (**B**) Mutation frequencies of HepG2, A3BKO, A3B-tGFP and A3B-KO + A3B-tGFP cells were calculated as (number of colonies with 6-TG)/(number of colonies without 6-TG × number of seeded cells × plating efficiency). (**C**) Radiation dose–survival curves of HepG2, A3BKO, Mock and A3B cells plated immediately after treatment with 2, 5 and 10 Gy of irradiation. Data are presented as means ± SEM of at least three experiments; ^*^P < 0.05. The same experiment was performed with two other independent clones of A3BKO cells and three independent clones of A3B-tGFP.

**Fig. 3.**
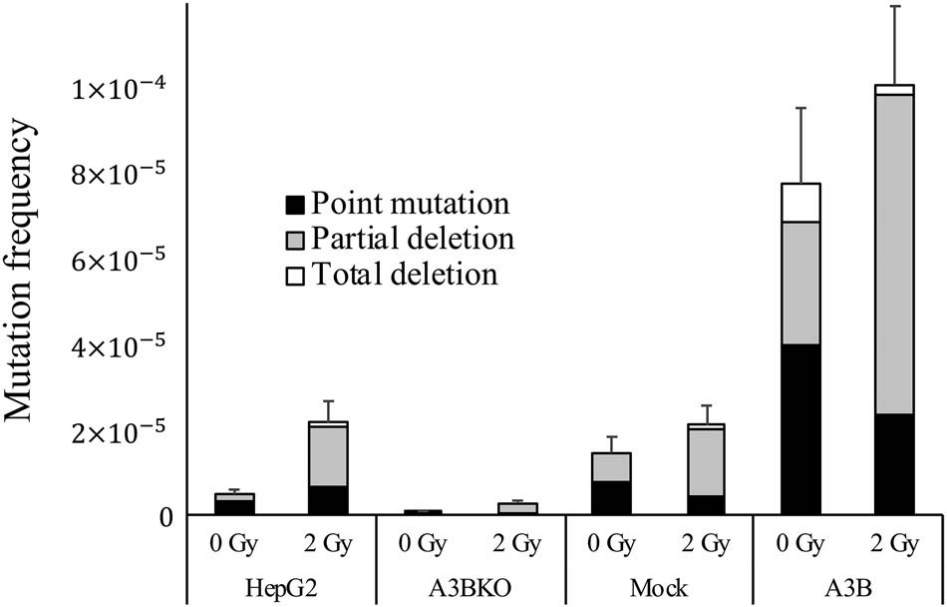
Knockout of APOBEC3B suppresses the increased mutation frequency after irradiation. Mutation frequency was measured using the HPRT assay. Cells were treated with HAT, irradiated with 2 Gy X-ray and incubated with 6-TG for 3 weeks after 7 days of phenotype expression. The percentages of point mutations, partial deletions and total deletions detailed in Table 1 are plotted. Data are presented as mean ± SEM.

While IR mainly causes indels, APOBEC3B causes GC to AT mutations on one side of the DNA strand [7, 18]. To assess the effect of APOBEC3B KO on spontaneous mutations in cancer cells, HAT-treated cells were cultured, and the number of cell divisions and the spontaneous mutation frequency of the *HPRT* gene were measured (Fig. 2A). The spontaneous mutation rate (determined from the slope of the line generated by regression analysis) was 1.89 ± 0.23 × 10^−6^ for HepG2 and 1.13 ± 0.10 × 10^−6^ for A3BKO, which was significantly lower (*P* < 0.05). The mutation rate of CRISPR-Cas9-treated control cells (1.50 ± 0.1 × 10^−6^) was not as low as that of A3BKO cells (*P* < 0.05). However, since high expression of APOBEC3B promotes mutation, cell death and cell morphological abnormalities [2], the mutation rate could not be determined using this assay, which takes ~1 month to measure. After passaging A3B-tGFP cells several times, cells did not form *HPRT*-mutation colonies expressing APOBEC3B (Supplementary Fig. 3, see online supplementary material). Instead, we determined the frequency of spontaneous mutation in HAT-treated cells after phenotype expression for 4 days. The frequency of spontaneous mutation was 1.23 × 10^−6^ for HepG2, 4.92 × 10^−7^ for A3BKO and 4.6 × 10^−6^ for A3B-tGFP (Fig. 2B). In A3BKO + A3B-tGFP cells, the mutation frequency was 6.12 × 10^−6^. Further, A3BKO cell survival decreased after treatment with 5 Gy IR, while survival of APOBEC3B stably expressing (A3B) cells increased slightly after 5 Gy IR (Fig. 2C). The increase in mutations caused by IR was analyzed using the HPRT assay after 1 week of phenotypic expression (Fig. 3). In HepG2 cells, 2 Gy IR increased the mutation frequency from 4.84 × 10^−6^ to 2.16 × 10^−5^. The mutation frequency in non-irradiated A3BKO cells was 6.46 × 10^−7^, and rose to only 2.70 × 10^−6^ when irradiated. Re-expression of APOBEC3B in KO cells increased their mutation frequency (before IR 6.38 × 10^−5^ and after 8.91 × 10^−5^) to the same extent as that observed in A3B cells before (7.76 × 10^−5^) and after (1.00 × 10^−4^) IR treatment (Fig. 3 and Supplementary Fig. 4, see online supplementary material). The ratios of radiation-induced mutations to spontaneous mutations were 4.44 (95% CI: 2.38–8.35) for HepG2 and 4.17 (95% CI: 1.97–8.90) for A3BKO cells; however, the ratios for partial deletions were 8.46 (95% CI: 4.04–17.8) for HepG2 and 3.64 (95% CI: 2.08–6.35) for A3BKO, with a trend toward a slight decrease for A3BKO. These results suggest that APOBEC3B expression made a small, but not significant, contribution to partial deletion mutations after IR. By contrast, APOBEC3B KO showed that endogenous levels of APOBEC3B expression have a significant effect on the spontaneous mutation rate and mutation frequency.

### Patterns of HPRT mutation after IR

APOBEC3B has cytidine deaminase activity against ssDNA, causing a series of GC to AT mutations [18], and can modify the genome in somatic cells *in vitro* and in high APOBEC3B-expressing cancer cells [11, 19]. To investigate whether the cytidine deaminase activity of APOBEC3B contributes to mutation of the *HPRT* gene by IR, the *HPRT* mutation pattern was examined. The presence or absence of nine *HPRT* exons was examined by PCR of *HPRT* mutants (Table 1). Before IR, large deletions were observed in 35.7, 46.2 and 48.6% of HepG2, Mock and A3B *HPRT*-mutant clones respectively, whereas 95.8% of the *HPRT*-mutant clones in A3BKO cells had large deletions. Single-base substitutions and short indels were less likely to occur in A3BKO cells. With 2 Gy IR, 70.0, 80.6, 77.5 and 85.0% of HepG2, Mock, A3B and A3BKO cells, respectively, had clones with large deletions. Hence, IR increased the rate of deletions in all cell lines except A3BKO. Next, to examine single-nucleotide substitutions or short indels, DNA sequence analysis was conducted using clones in which all *HPRT* exons were detected (Table 2, Supplementary Table 3, see online supplementary material). Some of the single-base substitution mutations detected were similar to those found in patients with Lesch–Nyhan disease, in which the HPRT enzyme is deficient, due to mutation of the gene [20]. Despite APOBEC3B expression, no particular trend toward single-base substitution mutations, such as GC to AT mutation, was observed. Nevertheless, APOBEC3B can also induce additional double-strand break (DSB) formation in response to DSBs [21], and this secondary DSB formation may explain the increase in mutation frequency.

**Table 1:** PCR analysis of *HPRT* mutants. Filled boxes indicate exons that could not be amplified by PCR. The number of clones in which each exon could not be detected is given, and the percentage this represents of the total number of clones is in parenthesis

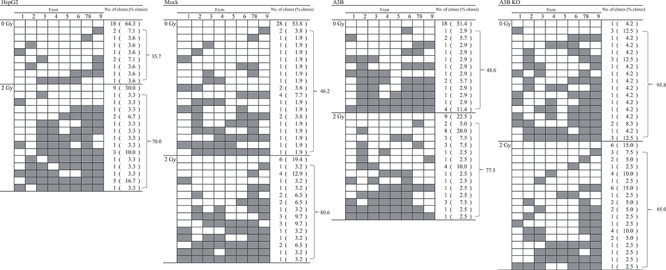

**Table 2:** Sequence analysis of *HPRT* point mutants. The mutated position in the exon ±2 bp is indicated and numbered according to its cDNA position. Affected bases and the 5′ and 3′ four bases flanking each side are shown

Cells	IR dose	Mutant	Position of mutation	Target sequence^a^ 5′ to 3′			Type of mutation	Amino acid change
HepG2	0 Gy	H 8–2, H 8–3, H 8–5	c.143	GAAC	G	TCTT	GC > TA	48 R > H
		H 8–1	c.551	AATT	C	CAGA	GC > TA	184 P > Q
		H 2–3, H2–6	c.610–1	tata	g	CATG	GC > AT	
		H8–6	c.623	GTCA	T	TAGT	AT > CG	208 I > S
		H 5–1	None					
	2 Gy	H2 5–9	c.114	TTCC	T	CATG	AT > GC	
			c.542	GGAT	T	TGAA	AT > TA	181 Y > F
			c.612	agCA	T	GTTT	AT > GC	
		H2 7–8	c.220	TAAA	T	TCTT	AT > GC	74 F > L
			c.346	AAAA	G	TAAT	GC > AT	116 V > I
			c.385–1	tcta	g	AATG	GC > TA	
		H2 7–9	c.302	ATCA	G	ACTG	GC > AT	101 R > K
		H2 6–20	c.402 + 1	GGAA	g	TAAT	GC > TA	
		H2 5–15	c.532 + 2	ACTg	t	aagt	AT > TA	
		H2 5–17	None					
Mock	0 Gy	C 8–1, C 8–2, C 8–6, C 8–7	c.238	GCTG	G	ATTA	GC > TA	80 D > Y
		C 3–27	c.580	CCTT	G	ACTA	GC > AT	194 D > N
		C 8–8	None					
	2 Gy	C2 4–7	c.95	GATT	T	GGAA	AT > GC	32 L > S
		C2 5–15	c.532 + 2	ACTg	t	aagt	AT > TA	
		C2 4–6	None					
A3B	0 Gy	G-3-1	c.59	CTTG	A	TTTA	AT > TA	20 D > V
		G 6–8	c.111	TTTA	T	TCCT	AT > CG	37 I > M
		G 3–8	c.319–1	acta	g	AATG	GC > CG	
		G 3–18	c.319–1	acta	g	AATG	GC > AT	
			c.346	AAAA	G	TAAT	GC > AT	116 V > I
		G 3–15	c.385–1	tcta	g	AATG	GC > CG	
			c.543	GATT	T	GAAA	AT > GC	
		G 4–3	c.403	aaag	G	ATAT	GC > AT	135 D > N
		G-3-2	c.520	TGGA	T	ATAA	AT > CG	174 Y > D
	2 Gy	G2 5–35	c.165	TGAA	G	GAGA	GC > CG	55 K > N
			c.188	ATTG	T	AGCC	AT > CG	63 V > G
		G2 5–10	c.166	GAAG	G	AGAT	GC > TA	56 E > ^*^
		G2 5–23	c.229	TGCT	G	ACCT	GC > TA	77 D > Y
		G2 4–3	c.384 + 1	AAAG	g	tatg	GC > AT	
		G2 4–1	c.428	ACAA	T	GCAG	AT > TA	143 M > K
A3B KO	0 Gy	3B 5–1	c.28–2	tttc	a	gATT	AT > TA	
	2 Gy	3B2 5–2	c.385–2	ttct	a	gAAT	AT > TA	
		3B2 5–28	c.402 + 1	GGAA	g	taag	GC > CG	

^a^Intron sequences are shown in lower case letters, whereas upper case indicates exon sequence.

^*^An asterisk indicates a terminal codon.

### APOBEC3B has a minor effect on DNA repair

**Fig. 4.**
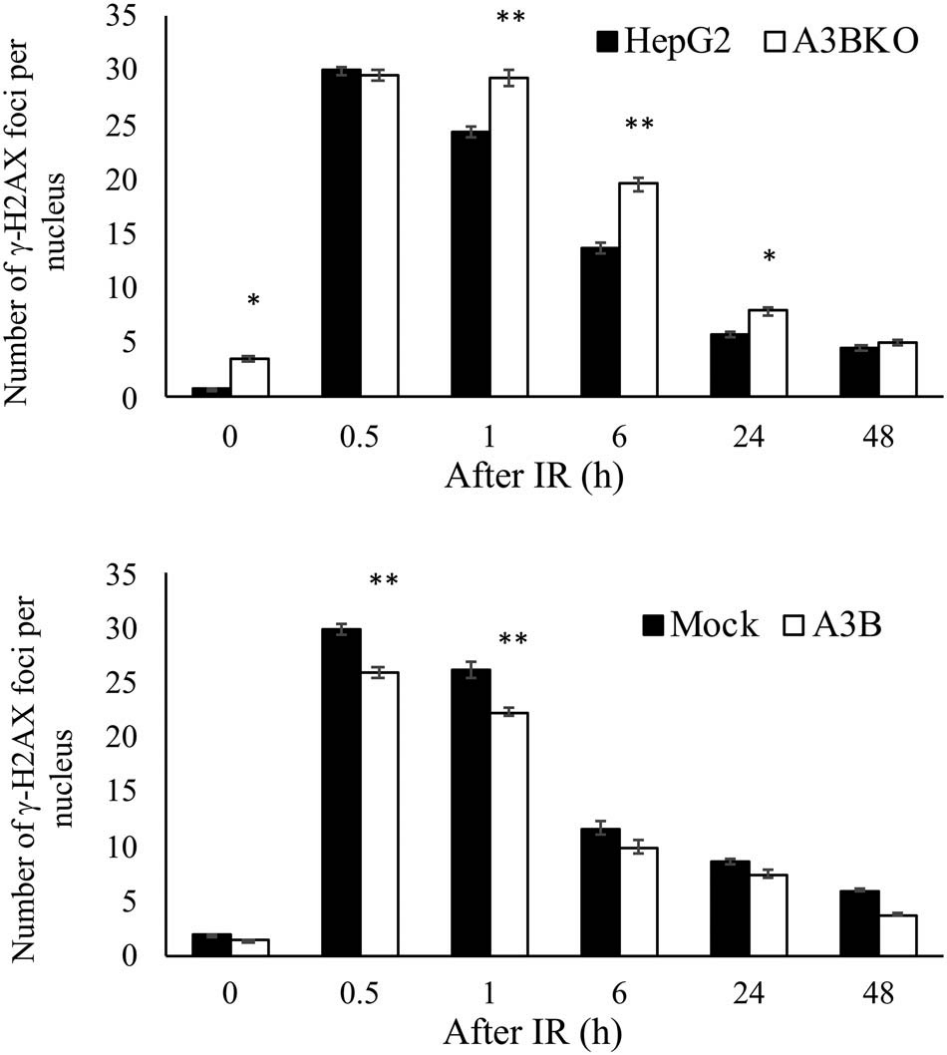
Effect of APOBEC3B on DNA damage repair after IR. HepG2, A3BKO, Mock and A3B cells were irradiated with 5 Gy X-ray, and stained with Alexa Fluor 488-conjugated anti-γ-H2AX Ab and Hoechst 33342 at the indicated times. Cell images were captured and the numbers of γ-H2AX foci per nucleus were analyzed using an Operetta CLS High-Content System. Data are presented as mean ± SEM; ^*^P < 0.05 vs control (HepG2); ^**^P < 0.01 vs control (HepG2 or Mock).

The involvement of APOBEC3B in post-irradiation mutations may be due to its function during DNA damage and repair, rather than because of an increase in mutations secondary to the induction of APOBEC3B expression. Therefore, to investigate whether APOBEC3B influences DNA damage repair, the number of γ-H2AX fluorescent signals (foci) per nucleus (as an index of DNA damage repair) was counted after 5 Gy IR, using an imaging analyzer (Fig. 4). The mean number of γ-H2AX foci in HepG2 cells was 29.9/nucleus 0.5 h after IR, which decreased to 24.3/nucleus after 1 h, whereas over the same time period the number of γ-H2AX foci in A3BKO cells barely changed (29.5/nucleus to 29.1/nucleus; Fig. 4). After 6 h, the numbers of γ-H2AX foci in HepG2 and A3BKO cells were 13.7/nucleus and 19.6/nucleus, respectively. Forced expression of APOBEC3B has been reported to induce DNA damage [10], leading to a decrease in cell viability and a delay in the cell cycle; however, in this study, constitutive expression of APOBEC3B did not appear to cause any apparent increase in DSBs or secondary DSBs after DNA damage. Conversely, knocking out APOBEC3B appeared to cause a slight delay in repair within 6 h after IR, with addition of APOBEC3B similarly leading to a slight acceleration of repair. Further, addition of APOBEC3B led to a rapid decrease in expression of monoubiquitinated-γ-H2AX following IR, implying that APOBEC3B does influence DNA damage repair (Supplementary Fig. 5, see online supplementary material). To further investigate the relationship between DNA damage and APOBEC3B, we examined colocalization of APOBEC3B and γ-H2AX. In cells expressing GFP fused to the C-terminus of APOBEC3B (A3B-AcGFP), A3B-AcGFP was uniformly distributed in the nucleus (Supplementary Fig. 6A, see online supplementary material). γ-H2AX expression was slightly increased both in cells with stable expression of A3B-AcGFP and in those with drug-inducible A3B-tGFP expression; however, there was no positive correlation between APOBEC3B and γ-H2AX expression levels in individual cells (Supplementary Fig. 6B–E). In addition, A3B-tGFP and γ-H2AX signals were not colocalized (Supplementary Fig. 6F).

Next, we examined the formation of γ-H2AX foci and the localization of A3B-AcGFP after IR (Supplementary Fig. 7). While both γ-H2AX and A3B-AcGFP signals were observed, they did not colocalize. Hence, our results do not support direct interaction of APOBEC3B with DSBs. We also found little or no increase in DSBs in cells stably expressing APOBEC3B (A3B or A3B-AcGFP) or with transient high expression (A3B-tGFP). These results suggest that, despite the increased mutation frequency due to APOBEC3B expression, the lack of increased homeostatic DNA damage and delayed repair may be due to the activation of an incidental DNA repair mechanism, rather than related to APOBEC3B function.

Overall, our data show that expression of the cytidine deaminase, APOBEC3B, led to an increase in the formation of short indels and partial deletions. Although irradiation induced APOBEC3B expression, the mutations caused by increased expression of APOBEC3B were relatively small. Nevertheless, APOBEC3B significantly influenced both spontaneous and radiation-induced mutations; therefore, studies to further elucidate the processes involved are warranted.

## DISCUSSION

This study demonstrated that APOBEC3B expression increased in a radiation dose-dependent and sustained manner after IR of the cancer cells tested. This increased expression of APOBEC3B may contribute to increased mutation rates at low irradiation dose rates and increased cancer risk from IR over long periods of time. APOBEC3B transcription is increased by an ATR/Chk1-dependent pathway in breast cancer, in addition to viral infection, PMA and interferon-α [22]. In the present study, we found that APOBEC3B expression gradually increased from 24 to 72 h after IR in all three cancer cell lines tested, and we speculate that the upregulated expression was probably the result of a DNA damage response.

Radiation-induced mutations are often deletions, but they do not have specific sequence or chromatin structure features. For example, mismatch repair (MMR)-deficient (*Mlh1−/−*) mice have an increased incidence of T-cell lymphoma when exposed to IR; however, whole genome analysis did not reveal a specific mutation pattern [23]. Nevertheless, Behjati *et al*. have shown that ionizing radiation generates short deletions with microhomology and balanced inversions in human radiation-associated secondary malignancies [2]. APOBEC3B is a known risk factor for carcinogenesis [19, 24], and high expression of APOBEC3B in tumors induces a characteristic mutation pattern called kataegis. In this study, APOBEC3B not only increased point mutations and indels, but also generated multiple mutations in the *HPRT* gene. Further, APOBEC3B is reported to generate additional, late-onset DSBs, originating from existing DSBs [21]; this secondary DSB formation may lead to the production of multiple mutations. Therefore, knocking out APOBEC3B would be expected to suppress indels and large deletions.

Increased expression of APOBEC3B in response to IR could contribute to acquisition of radiation resistance and new properties in cancer cells. HepG2–8960-R, a CRR cell line, has a high mutation frequency and high levels of APOBEC3B expression. APOBEC3B expression is not a requirement for radioresistance; however, it may promote mutations and acquisition of new properties. Moreover, APOBEC3B is reported to increase sensitivity to DNA damage response and repair inhibitors [18], and to contribute to the acquisition of resistance to anticancer drugs [12]. As well as increasing radiosensitivity, knocking out APOBEC3B also increases sensitivity to several anticancer drugs that target DNA (data not shown); however, the mechanism by which APOBEC3B deficiency affects drug sensitivity is unknown and is currently under investigation.

In this study, we clarified that IR induces APOBEC3B expression in cancer cells, and that APOBEC3B enhanced the radiation-induced mutation frequency. Therefore, APOBEC3B expression levels in cancer cells would be expected to influence the effects of radiation therapy and patient prognosis. Furthermore, the mutations caused by APOBEC3B were not only GC to AT variations, but also more diverse changes. Hence, the involvement of APOBEC3B in cancer cell mutations may require re-evaluation. Further studies are now required to fully elucidate the mechanisms underlying the enhancement of radiation-induced mutations by APOBEC3B.

## FUNDING

This work was supported by JSPS KAKENHI Grant Number 15 K19814 and 17 K16479.

## Supplementary Material

SUPPLEMENTARY_MATERIALS_and_METHODS_rraa069

S-Fig_1_rraa069

S-Fig_2AB_rraa069

S-Fig_2C_rraa069

S-Fig_3_rraa069

S-Fig_4_rraa069

S-Fig_5_rraa069

S-Fig_6A_rraa069

S-Fig_6B_rraa069

S-Fig_6C_rraa069

S-Fig_6DE_rraa069

S-Fig_6F_rraa069

S-Fig_7_rraa069

S-Table_1_rraa069

S-Table_2_rraa069

S-Table_3_rraa069

## ABBREVIATIONS

HPRT hypoxanthine-guanine phosphoribosyl transferase

IR irradiation

CRR clinically relevant radioresistant

## References

[1] Adewoye AB, Lindsay SJ, Dubrova YE et al. The genome-wide effects of ionizing radiation on mutation induction in the mammalian germline. Nat Commun 2015;6:6684. 25809527 10.1038/ncomms7684 PMC4389250

[2] Behjati S, Gundem G, Wedge DC et al. Mutational signatures of ionizing radiation in second malignancies. Nat Commun 2016;7:12605. 27615322 10.1038/ncomms12605 PMC5027243

[3] Kramer M, Stein B, Mai S et al. Radiation-induced activation of transcription factors in mammalian cells. Radiat Environ Biophys 1990;29:303–13. 2281136 10.1007/BF01210410

[4] Smirnov DA, Morley M, Shin E et al. Genetic analysis of radiation-induced changes in human gene expression. Nature 2009;459:587–91. 19349959 10.1038/nature07940 PMC3005325

[5] Kuwahara Y, Li L, Baba T et al. Clinically relevant radioresistant cells efficiently repair DNA double-strand breaks induced by X-rays. Cancer Sci 2009;100:747–52. 19215227 10.1111/j.1349-7006.2009.01082.x PMC11158180

[6] Conticello SG. The AID/APOBEC family of nucleic acid mutators. Genome Biol 2008;9:229. 10.1186/gb-2008-9-6-229 PMC2481415 18598372

[7] Nik-Zainal S, Alexandrov LB, Wedge DC et al. Mutational processes molding the genomes of 21 breast cancers. Cell 2012;149:979–93. 22608084 10.1016/j.cell.2012.04.024 PMC3414841

[8] Burns MB, Temiz NA, Harris RS. Evidence for APOBEC3B mutagenesis in multiple human cancers. Nat Genet 2013;45:977–83. 23852168 10.1038/ng.2701 PMC3902892

[9] Haradhvala NJ, Polak P, Stojanov P et al. Mutational strand asymmetries in cancer genomes reveal mechanisms of DNA damage and repair. Cell 2016;164:538–49. 26806129 10.1016/j.cell.2015.12.050 PMC4753048

[10] Taylor BJ, Nik-Zainal S, Wu YL et al. DNA deaminases induce break-associated mutation showers with implication of APOBEC3B and 3A in breast cancer kataegis. Elife 2013;2:e00534. 23599896 10.7554/eLife.00534 PMC3628087

[11] Akre MK, Starrett GJ, Quist JS et al. Mutation processes in 293-based clones overexpressing the DNA cytosine deaminase APOBEC3B. PLoS One 2016;11:e0155391. 27163364 10.1371/journal.pone.0155391 PMC4862684

[12] Law EK, Sieuwerts AM, LaPara K et al. The DNA cytosine deaminase APOBEC3B promotes tamoxifen resistance in ER-positive breast cancer. Sci Adv 2016;2:e1601737. 27730215 10.1126/sciadv.1601737 PMC5055383

[13] Gyorffy B, Surowiak P, Kiesslich O et al. Gene expression profiling of 30 cancer cell lines predicts resistance towards 11 anticancer drugs at clinically achieved concentrations. Int J Cancer 2006;118:1699–712. 16217747 10.1002/ijc.21570

[14] Nowarski R, Wilner OI, Cheshin O et al. APOBEC3G enhances lymphoma cell radioresistance by promoting cytidine deaminase-dependent DNA repair. Blood 2012;120:366–75. 22645179 10.1182/blood-2012-01-402123 PMC3398754

[15] Wang Y, Wu S, Zheng S et al. APOBEC3G acts as a therapeutic target in mesenchymal gliomas by sensitizing cells to radiation-induced cell death. Oncotarget 2017;8:54285–96. 28903341 10.18632/oncotarget.17348 PMC5589580

[16] Glaab WE, Tindall KR. Mutation rate at the hprt locus in human cancer cell lines with specific mismatch repair-gene defects. Carcinogenesis 1997;18:1–8. 9054582 10.1093/carcin/18.1.1

[17] Kuwahara Y, Roudkenar MH, Urushihara Y et al. Clinically relevant radioresistant cell line: A simple model to understand cancer radioresistance. Med Mol Morphol 2017;50:195–204. 29067564 10.1007/s00795-017-0171-x

[18] Nikkila J, Kumar R, Campbell J et al. Elevated APOBEC3B expression drives a kataegic-like mutation signature and replication stress-related therapeutic vulnerabilities in p53-defective cells. Br J Cancer 2017;117:113–23. 28535155 10.1038/bjc.2017.133 PMC5520199

[19] Zou J, Wang C, Ma X et al. APOBEC3B, a molecular driver of mutagenesis in human cancers. Cell Biosci 2017;7:29. 28572915 10.1186/s13578-017-0156-4 PMC5450379

[20] Jinnah HA, De Gregorio L, Harris JC et al. The spectrum of inherited mutations causing HPRT deficiency: 75 new cases and a review of 196 previously reported cases. Mutat Res 2000;463: 309–26. 11018746 10.1016/s1383-5742(00)00052-1

[21] Shimizu A, Fujimori H, Minakawa Y et al. Onset of deaminase APOBEC3B induction in response to DNA double-strand breaks. Biochem Biophys Rep 2018;16:115–21. 30417129 10.1016/j.bbrep.2018.10.010 PMC6216020

[22] Kanu N, Cerone MA, Goh G et al. DNA replication stress mediates APOBEC3 family mutagenesis in breast cancer. Genome Biol 2016;17:185. 27634334 10.1186/s13059-016-1042-9 PMC5025597

[23] Daino K, Ishikawa A, Suga T et al. Mutational landscape of T-cell lymphoma in mice lacking the DNA mismatch repair gene Mlh1: No synergism with ionizing radiation. Carcinogenesis 2019;40:216–24. 30721949 10.1093/carcin/bgz013

[24] Kuong KJ, Loeb LA. APOBEC3B mutagenesis in cancer. Nat Genet 2013;45:964–5. 23985681 10.1038/ng.2736 PMC3965181

