# Supplementary material for: Involvement of APOBEC3B in mutation induction by irradiation
Source: J Radiat Res. 2020 Sep 3;61(6):819–27. doi: 10.1093/jrr/rraa069 (PMC7674755; doi:10.1093/jrr/rraa069)
Supplement: SUPPLEMENTARY_MATERIALS_and_METHODS_rraa069 [file supplementary_materials_and_methods_rraa069.doc]

**SUPPLEMENTARY MATERIALS and METHODS**

**PCR of KO cells after genome editing**

PCR of the intron 2/exon 3 border of APOBEC3B using PrimeSTARDNA MAX polymerase and 5’-GACACACTGCCCTTCCAGAT-3’ as the forward primer, and 5’-TACTGAGGCTTGAAATACAC-3’ or 5’-GAGGAAGCACATTTCTGCGTGG-3’ as the reverse primer.

**Plasmid vector and transfection**

The pcDNA5.2-APOBEC3B (A3B) vector was constructed by inserting APOBEC3B-ORF (#RG222712; Origene, Rockville, MD, USA) into pcDNA5.2 (Thermo Fisher Scientific, Waltham, MA, USA). A3B expressing cell line (A3B) was transfected with A3B vector into HepG2, and selected with Blastsidin S (Nacalai Tesque, Inc.). TurboGFP(tGFP)-tagged APOBEC3B expressing cell lines (A3B-tGFP or A3BKO + A3B-tGFP) were transfected with #RG222712 into HepG2 or A3BKO cells, and selected with G418 (Nacalai Tesque, Inc.).

APOBEC3B-AcGFP and AcGFP-APOBEC3B vectors were constructed by inserting APOBEC3B-ORF and a linker into pAcGFP1-N1 and pAcGFP-C1, respectively. The plasmids were transfected into HepG2 using FuGENE-HD (Promega), and stably expressing cell lines were selected with G418.

Tet-on APOBEC3B-tGFP (tetA3B-tGFP) vector was constructed by inserting APOBEC3B-tGFP from tGFP-tagged APOBEC3B (#RG222712; Origene) and a linker into the pTetOne vector (Takara Bio, Inc., Shiga, Japan). The plasmid and linear hygromycin marker together were transfected into HepG2 using Xfect Transfection (Takara Bio, Inc.), and cells were selected with hygromycin (Nacalai Tesque, Inc.). A3B-tGFP expression was induced with doxycycline (Dox; FUJIFILM Wako Pure Chemical Corporation, Osaka, Japan) at 〜30 ng/mL final concentration for 48 h.

**Western blotting**

To investigate γ-H2AX, HepG2, A3BKO, Mock, and A3B cells were irradiated with 5 Gy X-ray. The cells were collected at the indicated time points and lysed prior to immunoblotting with an anti-histone H2A.X antibody (phospho S139; [EP854(2)Y] ChIP Grade; Abcam PLC, Cambridge, UK) or with an anti-actin pAb.

**HPRT exon PCR conditions**

The PCR conditions for primer set A were as follows: 15 s at 98˚C, followed by 35 cycles for 10 s at 98˚C and 30 s at 68˚C, and one cycle for 7 min at 72˚C. Conditions for primer set B were as follows: 2 min at 95˚C, followed by 35 cycles for 30 s at 95˚C, 30 s at 61˚C, and 1 min at 72˚C, and then one cycle for 5 min at 72˚C. All the exon sequences of the HPRT locus were amplified by PCR.

**Antibodies used for western blotting**

The membrane was blocked with Blocking One (Nakarai tesque) and then incubated with an anti-APOBEC3B mAb (EPR18138; Abcam), an anti-APOBEC3C pAb (Proteintech Group, Inc., Rosemont, IL, USA), an anti-APOBEC3D pAb (Signalway Antibody, LLC, College Park, MD, USA), an anti-APOBEC3F pAb (GeneTex, Inc., Irvine, CA, USA), or an anti-APOBEC3G pAb (Bioss Antibodies, Boston, MA, USA).

**FIGURE LEGENDS**

**SUPPLEMENTARY FIGURE 1** High expression of APOBEC3B in HepG2-8960-R.

HepG2 and HepG2-8960-R cell pellets were lysed with SDS sample buffer to perform immunoblotting using anti-APOBEC3B and actin (internal control) antibodies.

**SUPPLEMENTARY FIGURE 2** CRISPR/Cas9-mediated APOBEC3B knockout gene.

APOBEC3B KO cell lines (#3 and #15) were established by CRISPR/Cas9-mediated genome editing of HepG2. (A) PCR was used to check mutation of the APOBEC3B gene in extracted genomic DNA from three transformants (#3, #4, and #15). The following primers were used: 5’-GACACACTGCCCTTCCAGAT-3’ and 5’-TACTGAGGCTTGAAATACAC-3’ to amplify bases c.230–335 to 250, and 5’-GACACACTGCCCTTCCAGAT-3’ and 5’-GAGGAAGCACATTTCTGCGTGG-3’ to amplify bases c.230–335 to 271. Transformants #3 and #15 have a deletion across the intron 2/exon 3 border of the APOBEC3B gene. SOX21 was used as an internal control. (B) APOBEC3B protein was not detectable in transformants #3 and #15.

(C) APOBEC3 family members expression in HepG2, A3B-tGFP, A3BKO and A3BKO+A3BtGFP cells. HepG2 and each 2 clones of A3B-tGFP, A3BKO and A3BKO+A3BtGFP cells were lysed and examined for APOBEC3B, APOBEC3C, APOBEC3D, APOBEC3F, APOBEC3G and actin as internal control by immunoblotting as Fig. 1B.

**SUPPLEMENTARY FIGURE 3** APOBEC3B-tGFP expression affects mutation rate.

Mutation rates were measured using the HPRT assay. APOBEC3B-tGFP(A3B-tGFP) constant expression cells were pre-incubated with growth medium containing HAT supplement for 1 month to eliminate HPRT-mutant cells, and then incubated with growth medium containing 6-TG for 3 weeks. Mutation rate (mutations/cell/generation) was then obtained by plotting the observed mutant frequency as a function of population doubling and calculating the slope by linear regression analysis. A colony having less than 10% of cells in which GFP fluorescence was observed was defined as a low GFP intensity group. Other groups were normal GFP intensity groups.

**SUPPLEMENTARY FIGURE 4** Mutation fractions of A3B-KO+A3B-tGFP cells were calculated as (number of colonies with 6-TG)/(number of colonies without 6-TG × number of seeding cells × plating efficiency).

**SUPPLEMENTARY FIGURE 5** APOBEC3B decreased monoubiquitinated -γ-H2AX.

HepG2, A3BKO, Mock, and A3B cells were irradiated with an X-ray dose of 5 Gy. Cells were collected at the indicated times and lysed with SDS sample buffer. Immunoblotting was performed using anti-γ-H2AX and anti-actin Abs.

**SUPPLEMENTARY FIGURE 6** APOBEC3B induced γ-H2AX foci.

(A) HepG2 were transfected with expression vector containing AcGFP1 linked to the C- or N-terminal of APOBEC3B (A3B-AcGFP and AcGFP-A3B, respectively). Live cells were stained with Hoechst 33342 for 10 min, and cell images were captured using an Operetta CLS High-Content System. (B, C) Doxycycline (Dox)-inducible APOBEC3B-tGFP-expressing (tet A3B-tGFP) cells were cultured in medium containing Dox at a final concentration of 3, 10, or 30 ng/mL for 48 h. Cell were fixed, permeabilized, and stained with anti-γ-H2AX Ab and Alexa Fluor 594-conjugated secondary Ab. Cell images were captured using an Operetta CLS High-Content System. (C) The number of γ-H2AX foci per nucleus in tet A3B-tGFP cells with 0, 3, 10, or 30 ng/mL of Dox. Data are presented as mean ± SD. (D, E) Stable APOBEC3B-expressing cells (A3B-AcGFP), transient APOBEC3B-expressing cells, and control cells (HepG2) were stained, and γ-H2AX foci were counted as above. (E) The number of γ-H2AX foci per nucleus. Data were analyzed using the Welch’s *t-*test and presented as mean ± SEM. **, *p* < 0.01 *vs*. control. (F) tet A3B-tGFP cells were incubated with 30 ng/mL of Dox for 48 h, and stained with anti-γ-H2AX Ab and Hoechst 33342. Confocal cell images were captured using an Operetta CLS High-Content System. The white arrow shows an APOBEC3B signal, and the red arrow shows a γ-H2AX focus. Scale bar is 10 µm.

**SUPPLEMENTARY FIGURE 7** APOBEC3B-tGFP did not colocalize with γ-H2AX after irradiation.

A3B-AcGFP stably expressing cells were selected with G418. Cells were irradiated with an X-ray dose of 5 Gy, fixed at the indicated times, and stained with Alexa Fluor 488-conjugated anti-γ-H2AX mAb and Hoechst 33342. Cell images were captured using an Operetta CLS High-Content System.

**SUPPLEMENTARY TABLE 1** qPCR primer sets. These are the qPCR primers for the SYBR GREEN method.

**SUPPLEMENTARY TABLE 2** HPRT Assay primer sets. There are two different PCR primer sets with different amplification positions.

**SUPPLEMENTARY TABLE 3** Sequence analysis of HPRT small deletion or insertion.
