# Supplementary figures and images for: Involvement of APOBEC3B in mutation induction by irradiation
Source: J Radiat Res. 2020 Sep 3;61(6):819–27. doi: 10.1093/jrr/rraa069 (PMC7674755; doi:10.1093/jrr/rraa069)

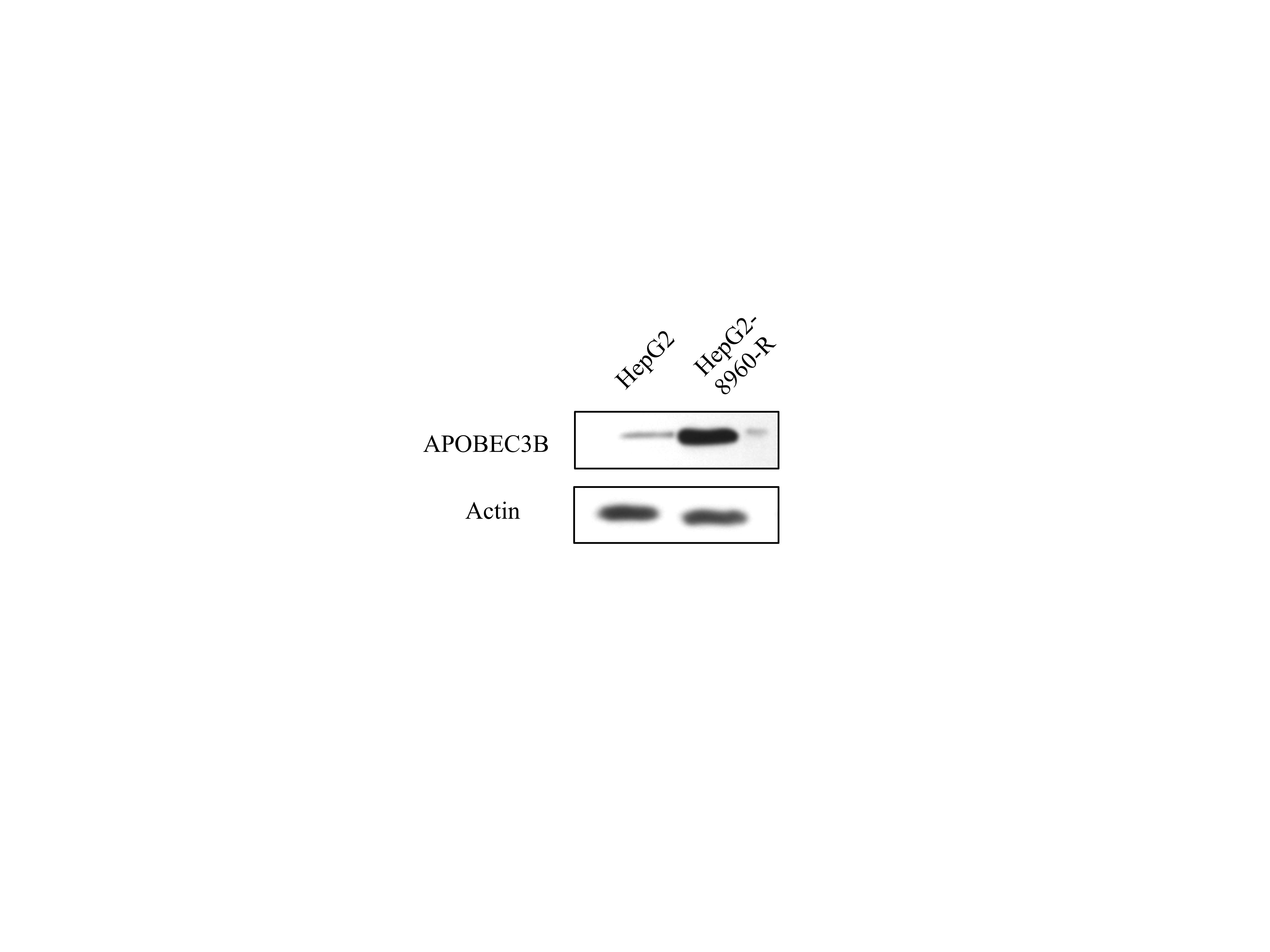

Supplement: S-Fig_1_rraa069 [file s-fig_1_rraa069.png]

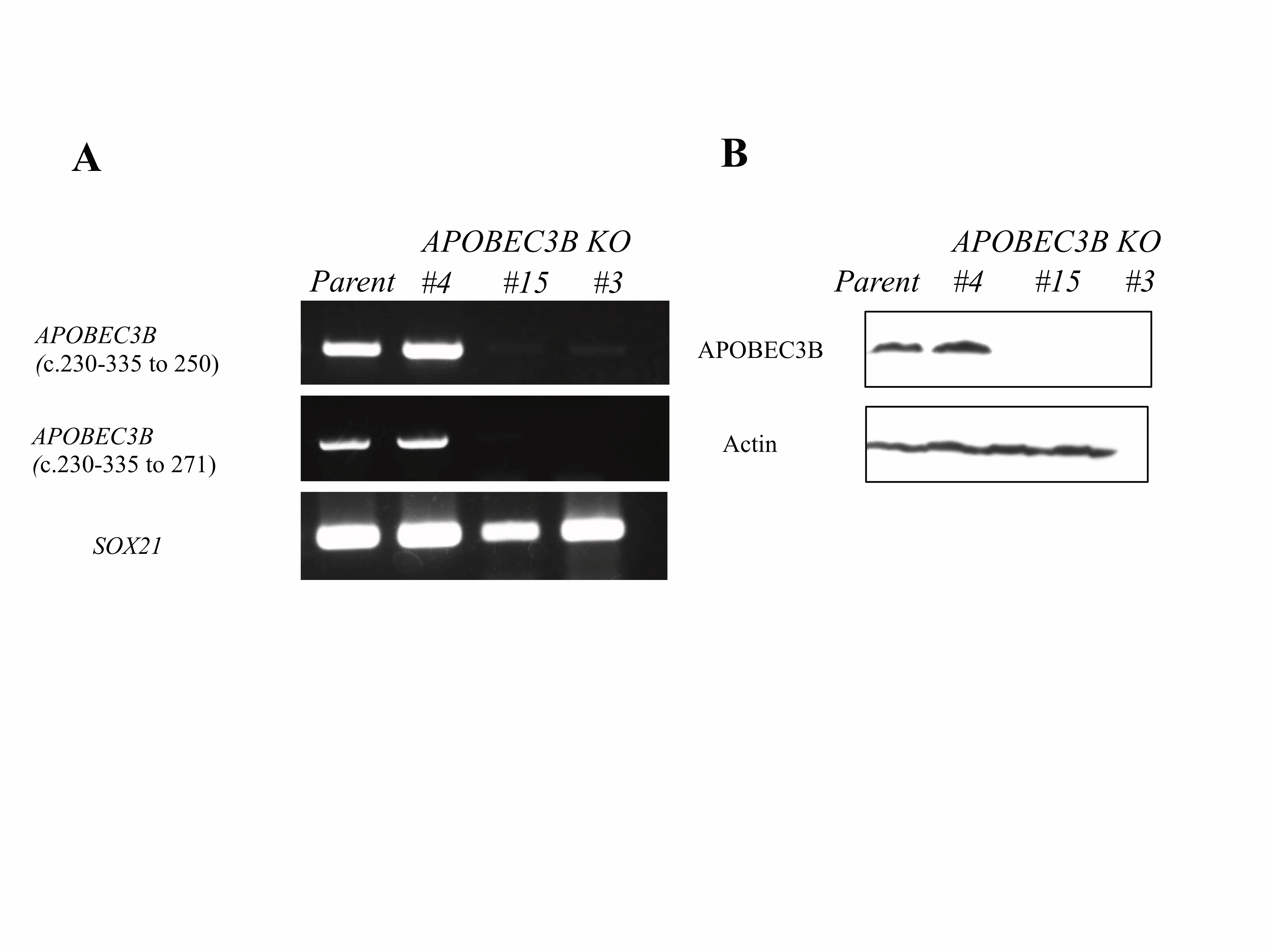

Supplement: S-Fig_2AB_rraa069 [file s-fig_2ab_rraa069.png]

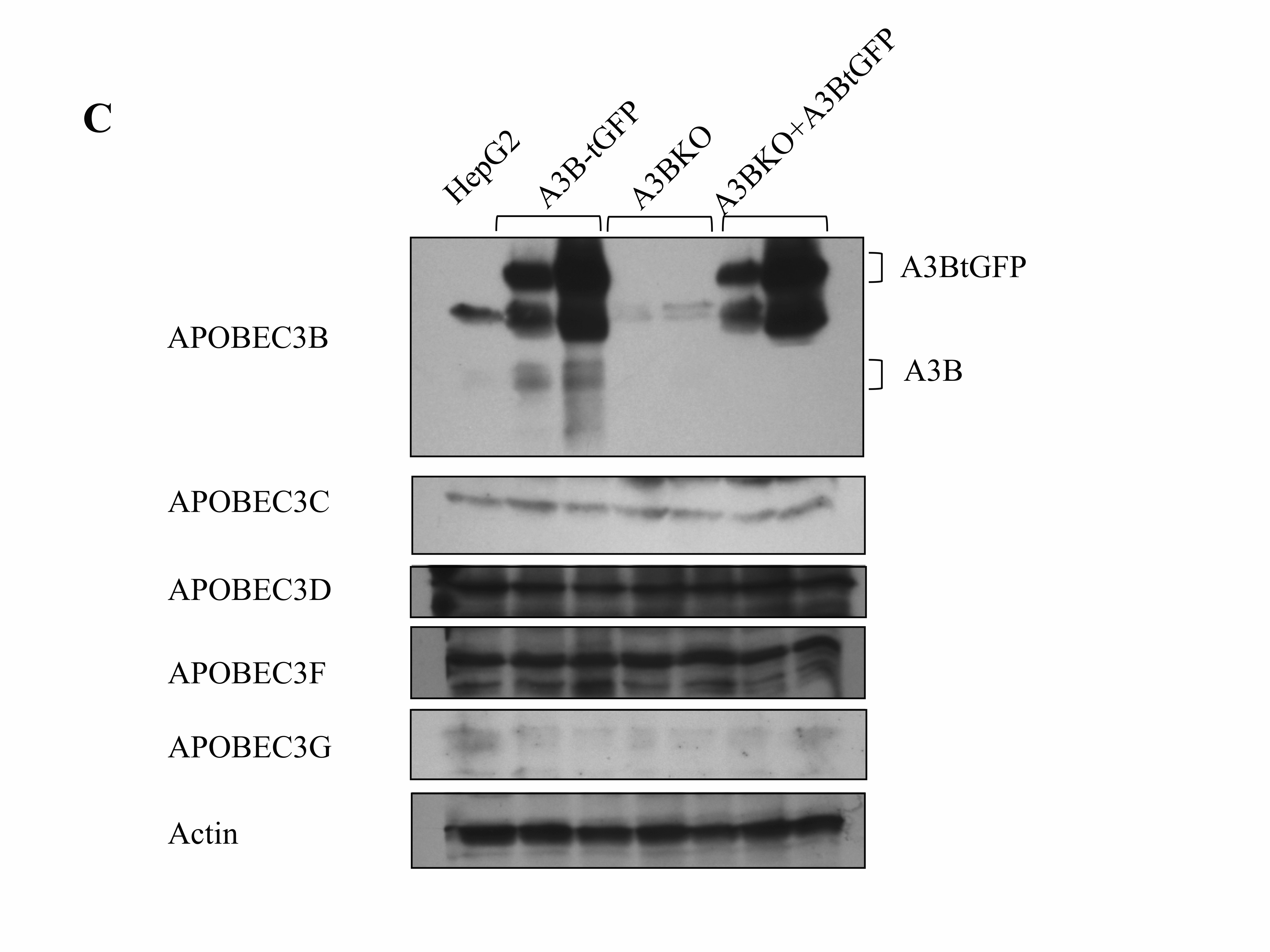

Supplement: S-Fig_2C_rraa069 [file s-fig_2c_rraa069.png]

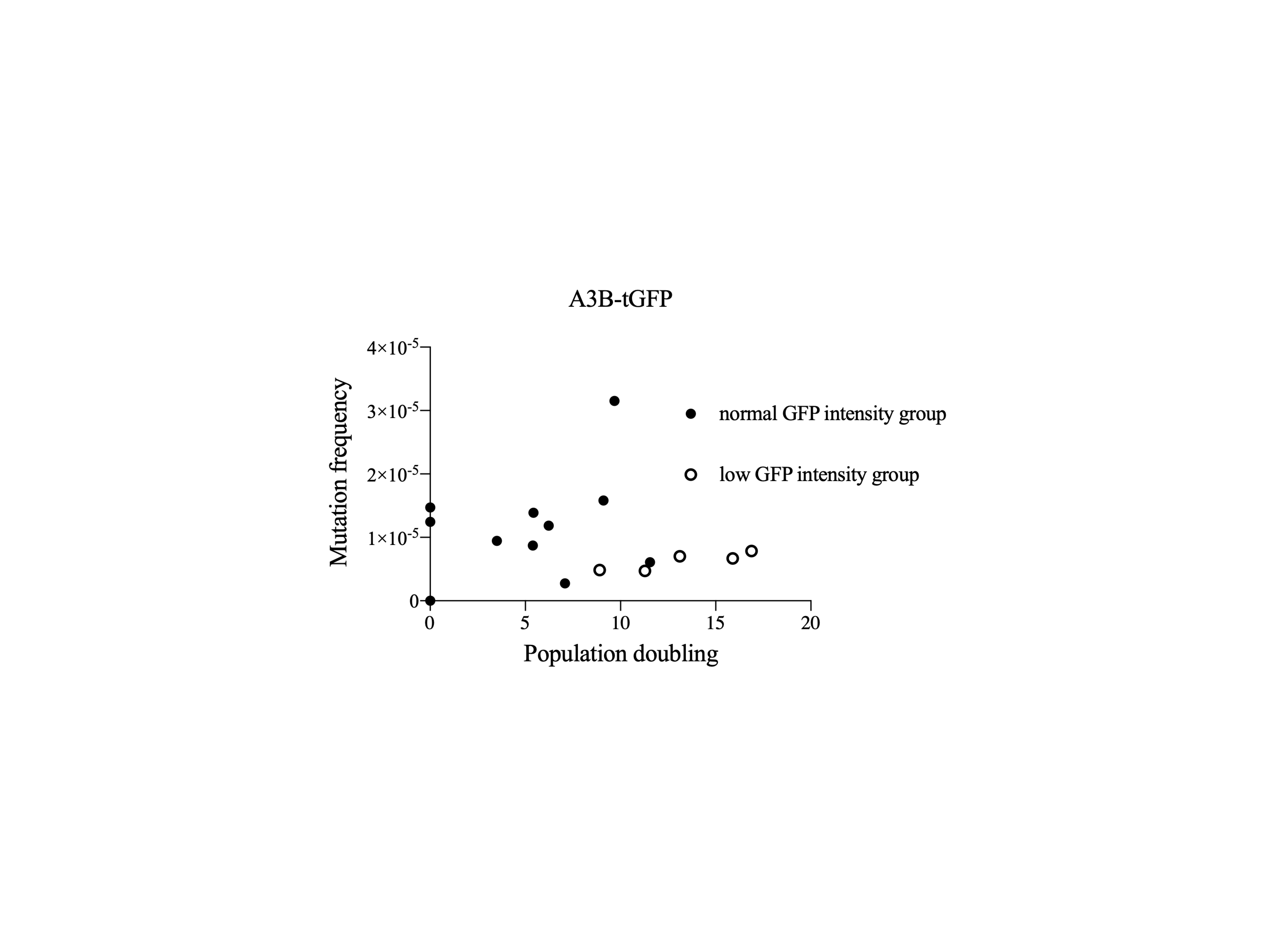

Supplement: S-Fig_3_rraa069 [file s-fig_3_rraa069.png]

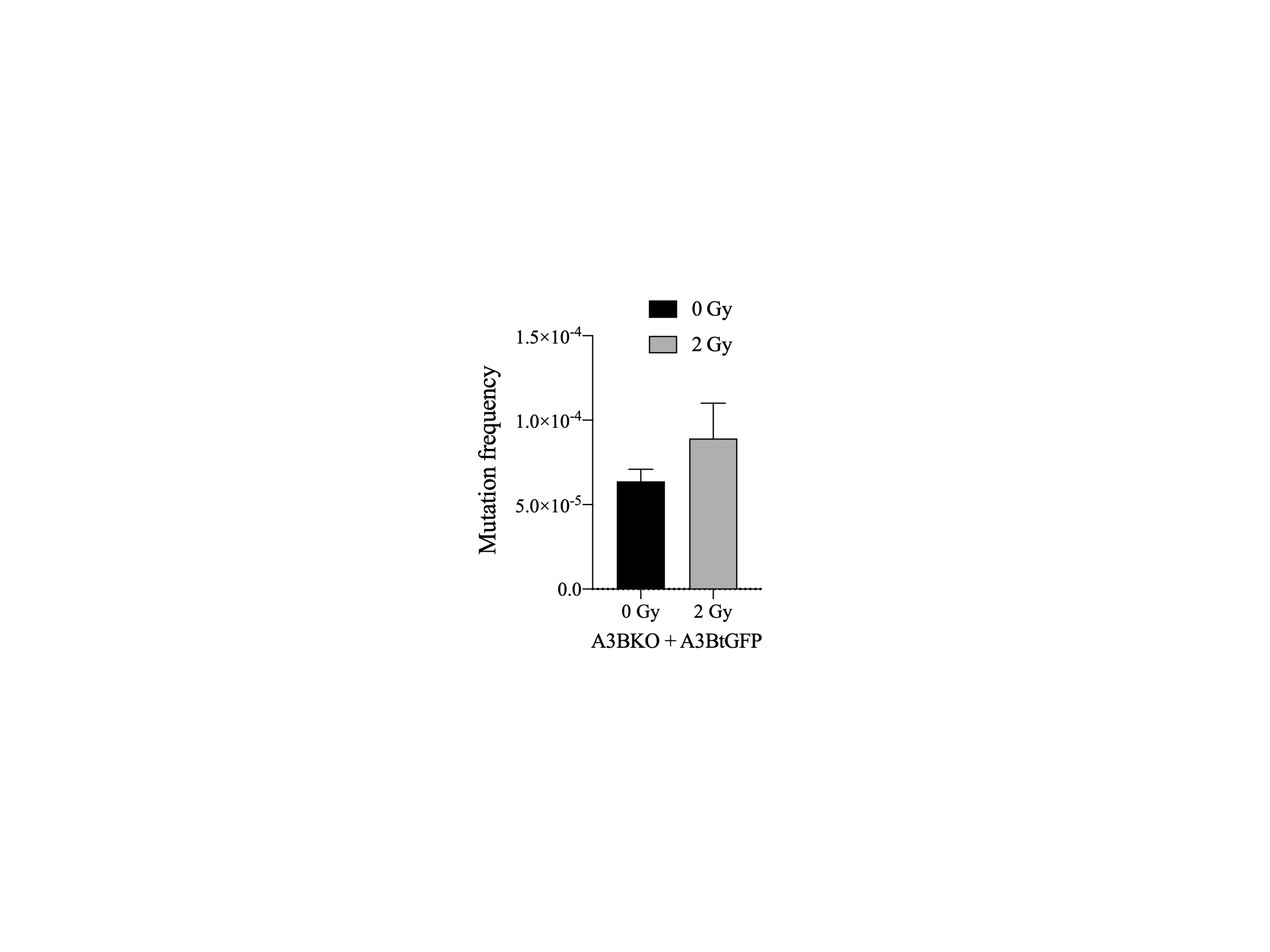

Supplement: S-Fig_4_rraa069 [file s-fig_4_rraa069.png]

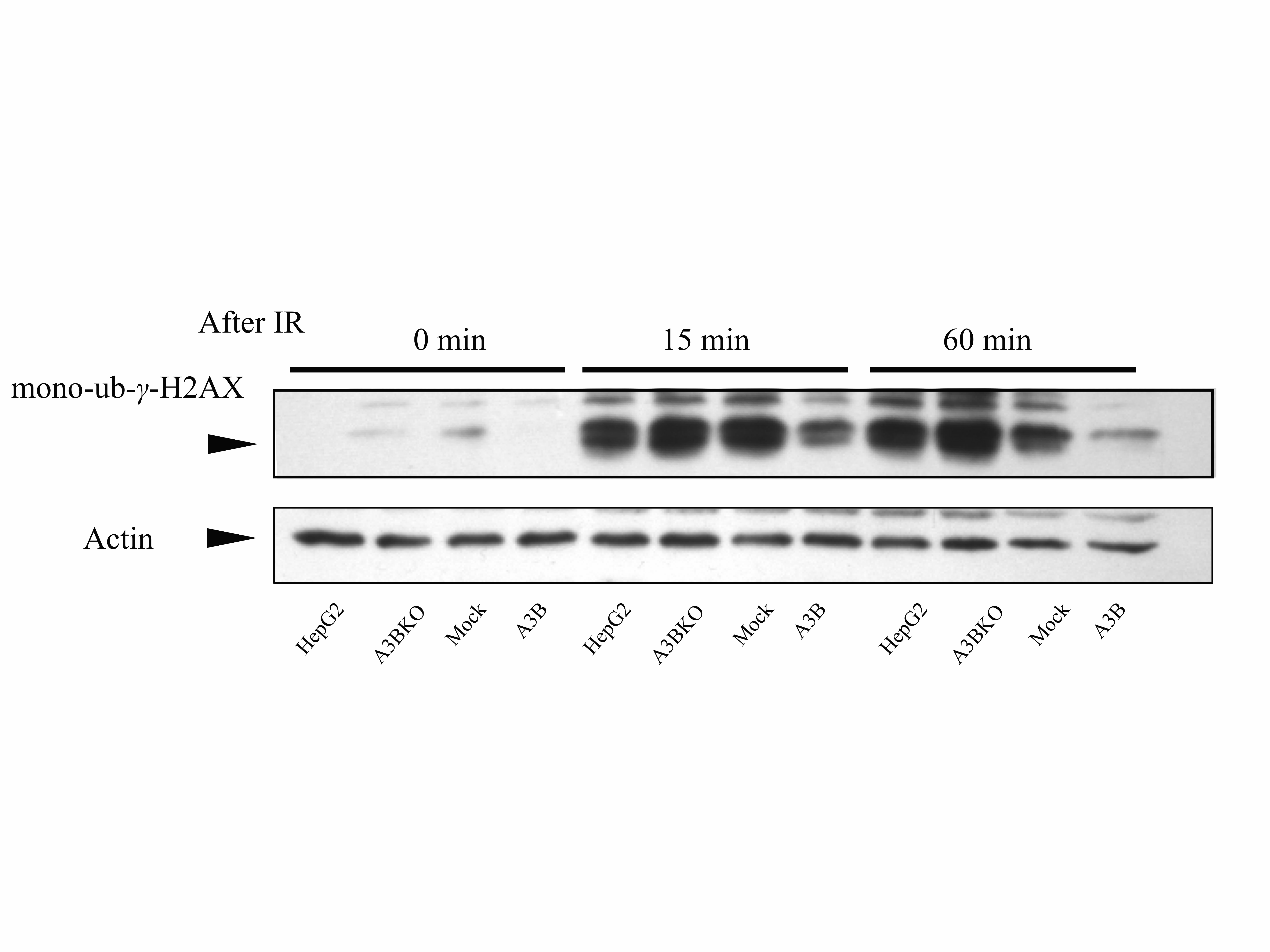

Supplement: S-Fig_5_rraa069 [file s-fig_5_rraa069.png]

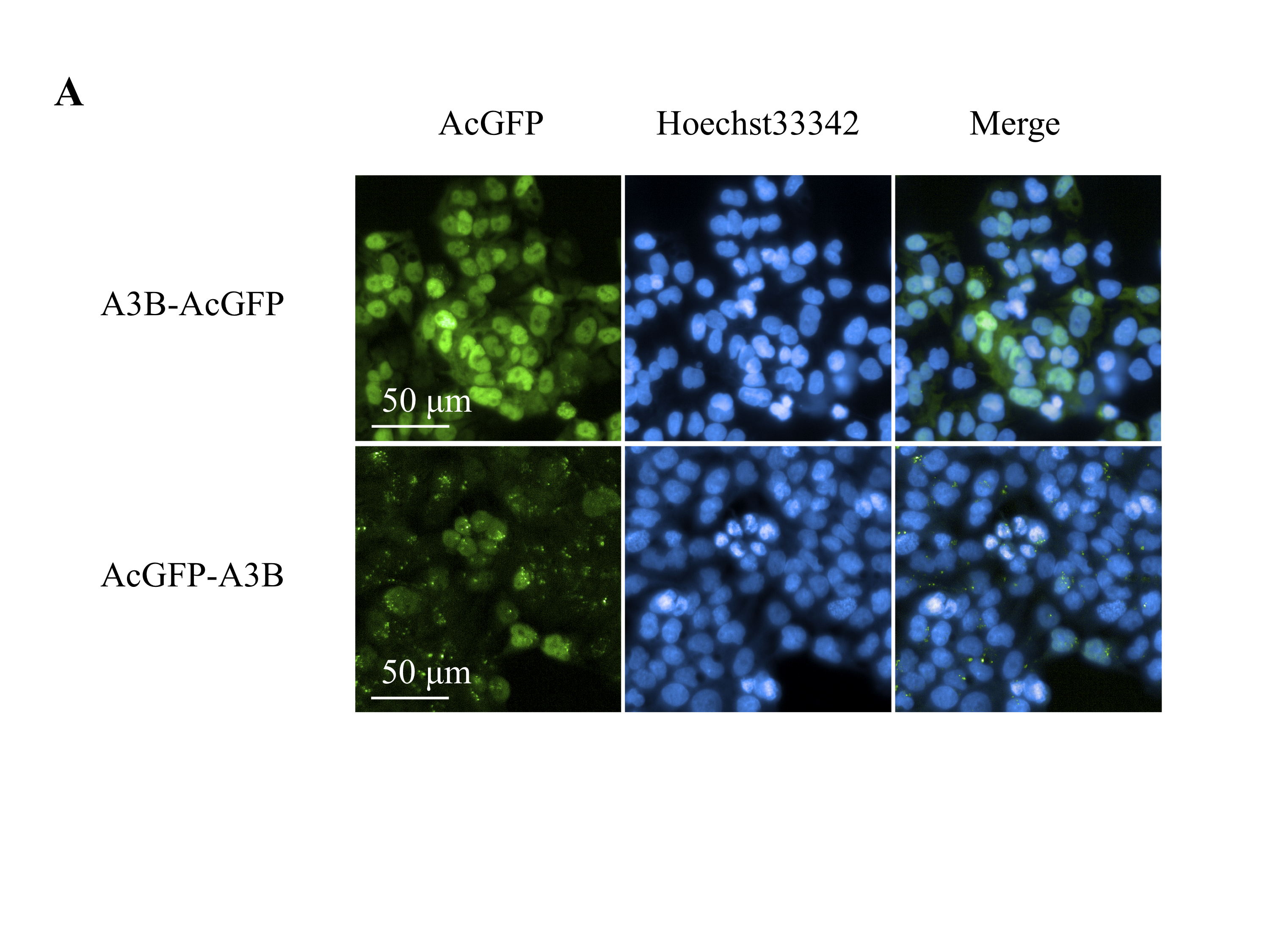

Supplement: S-Fig_6A_rraa069 [file s-fig_6a_rraa069.png]

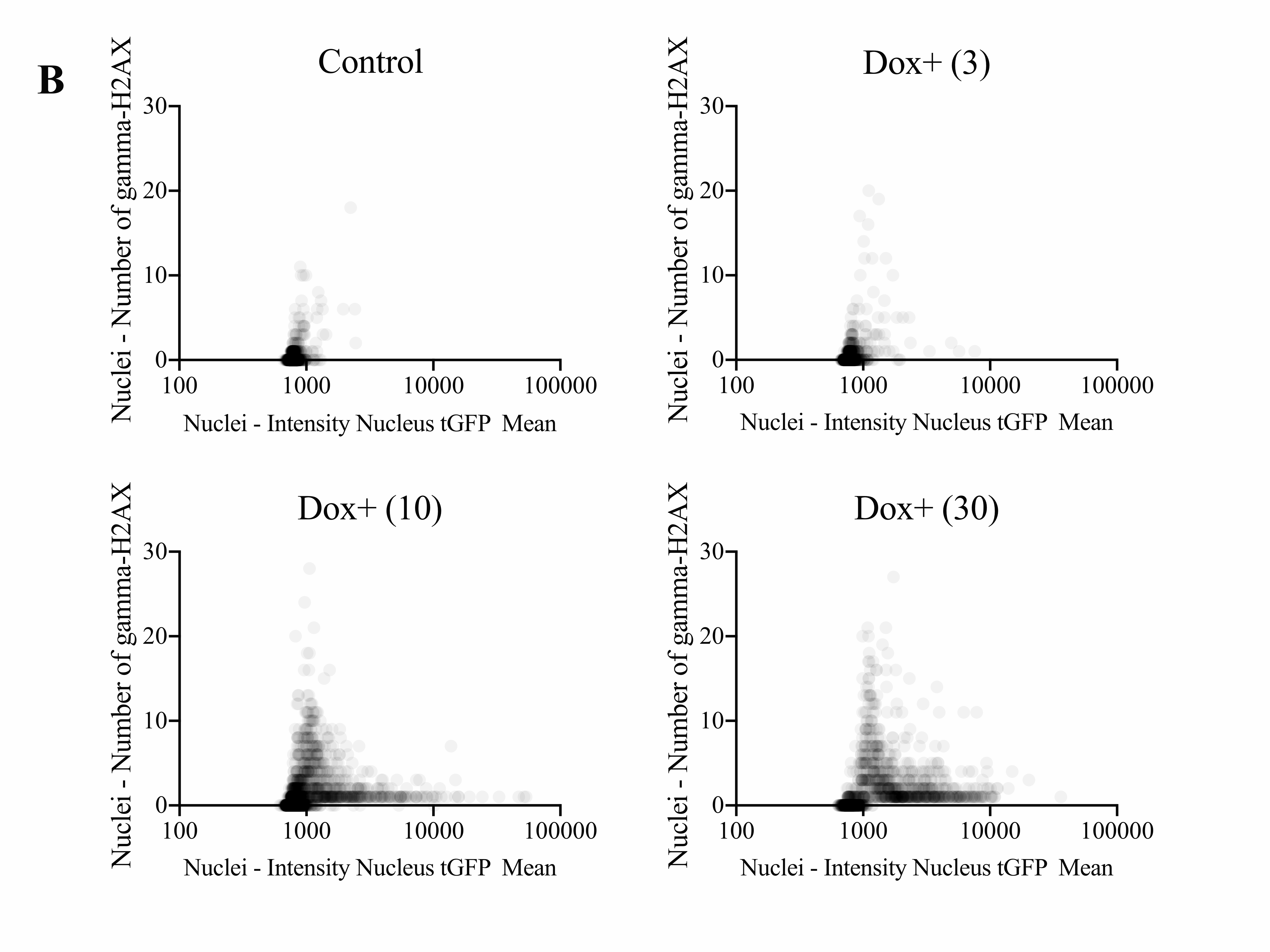

Supplement: S-Fig_6B_rraa069 [file s-fig_6b_rraa069.png]

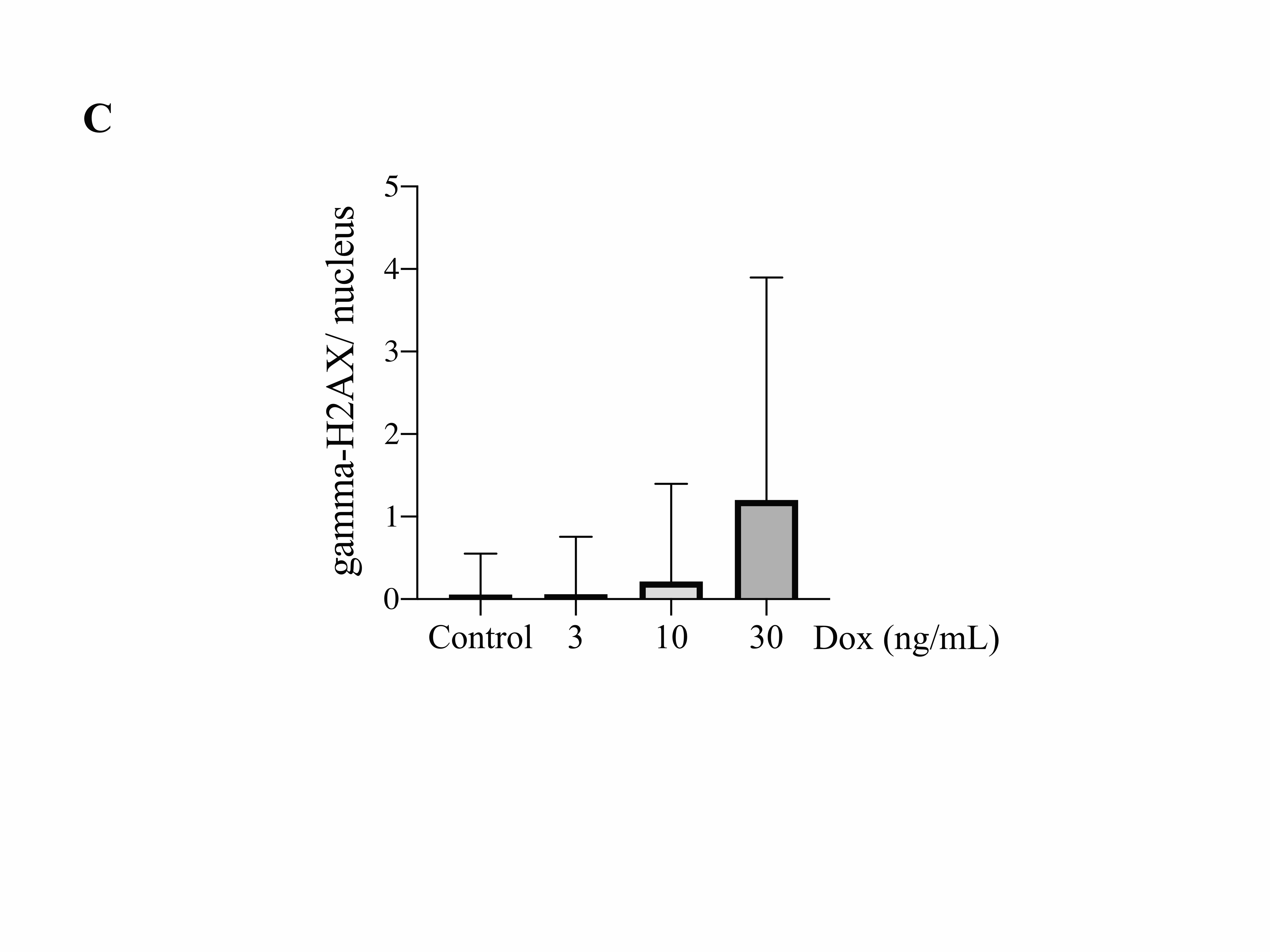

Supplement: S-Fig_6C_rraa069 [file s-fig_6c_rraa069.png]

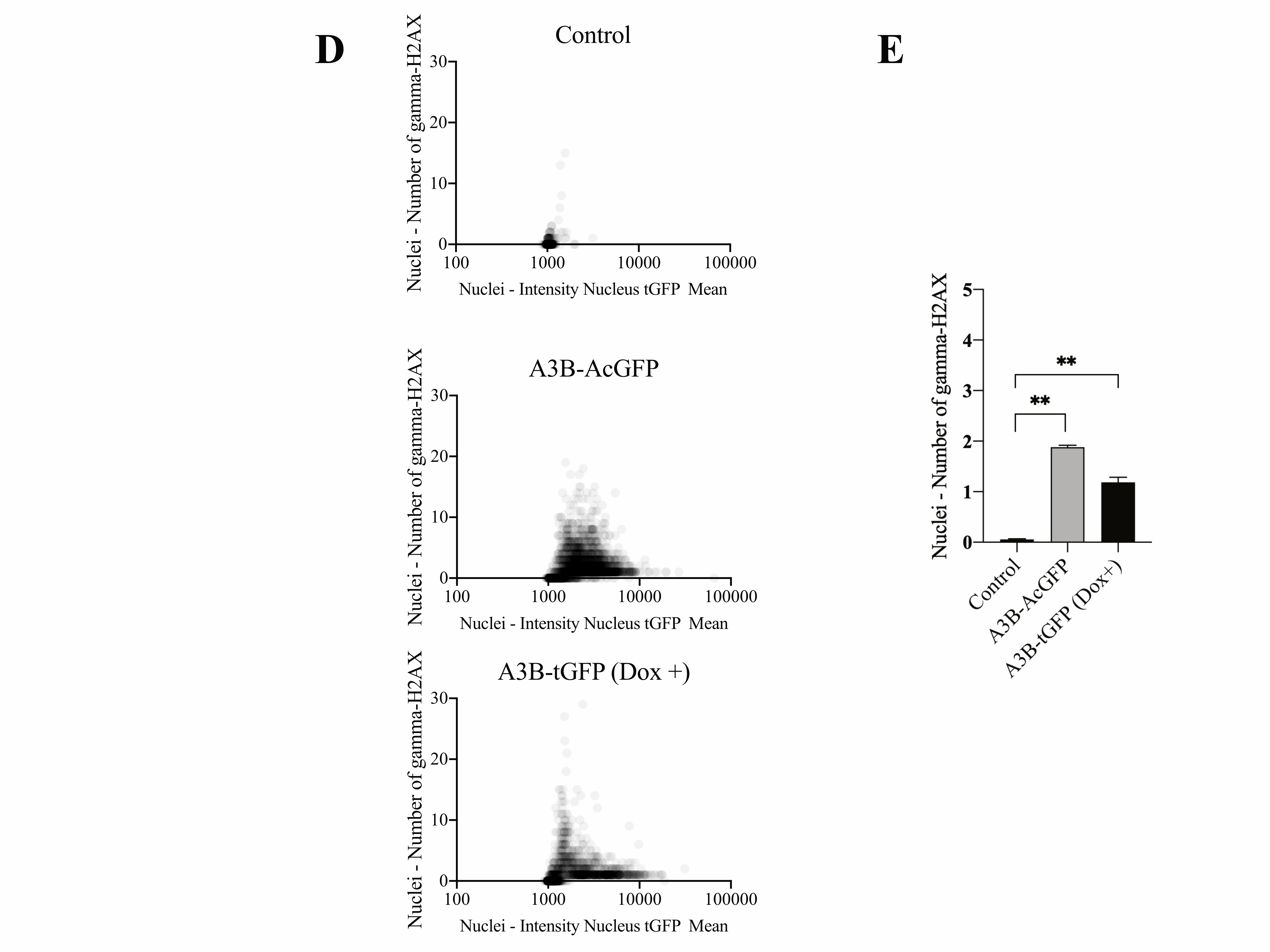

Supplement: S-Fig_6DE_rraa069 [file s-fig_6de_rraa069.png]

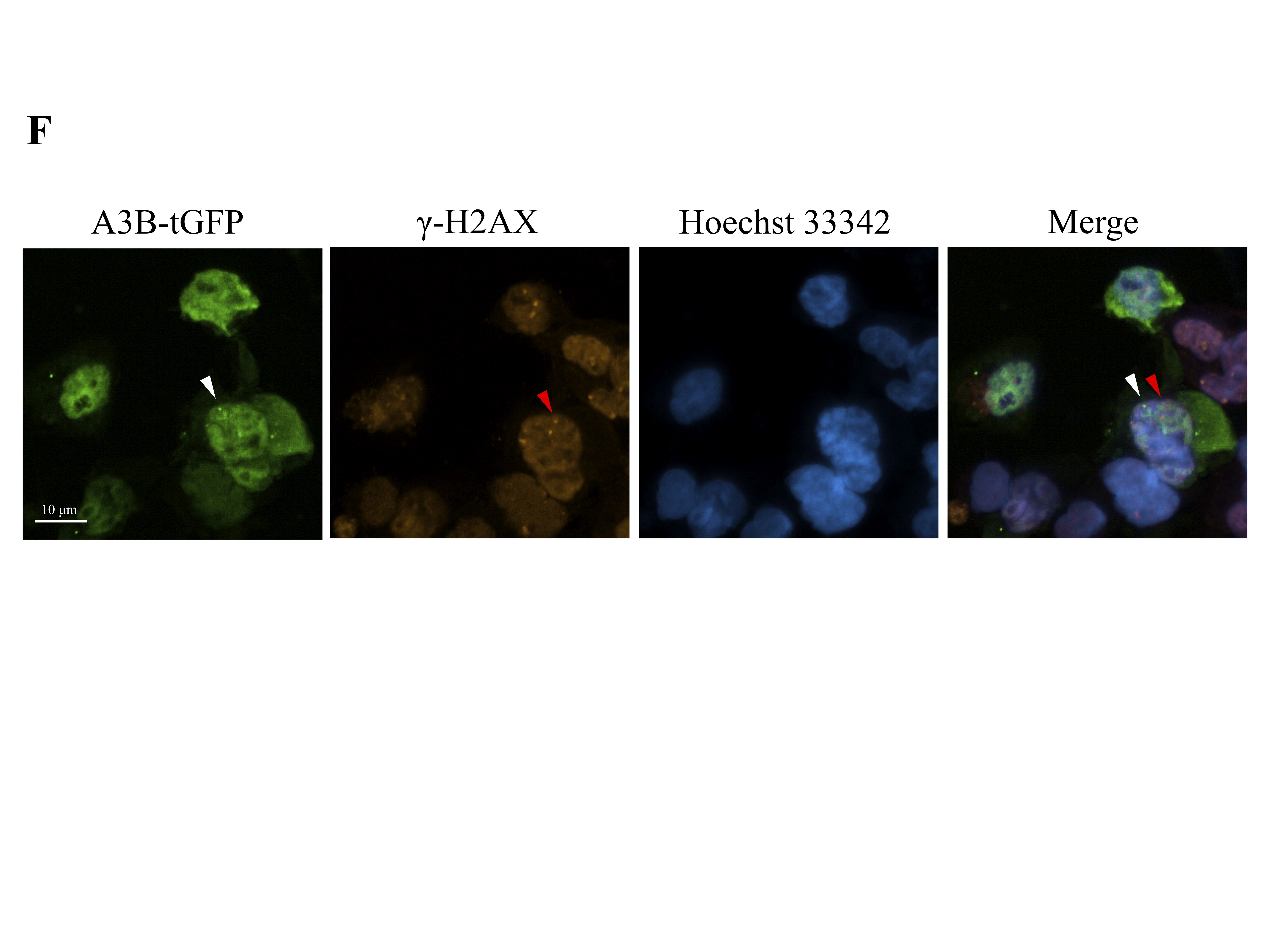

Supplement: S-Fig_6F_rraa069 [file s-fig_6f_rraa069.png]

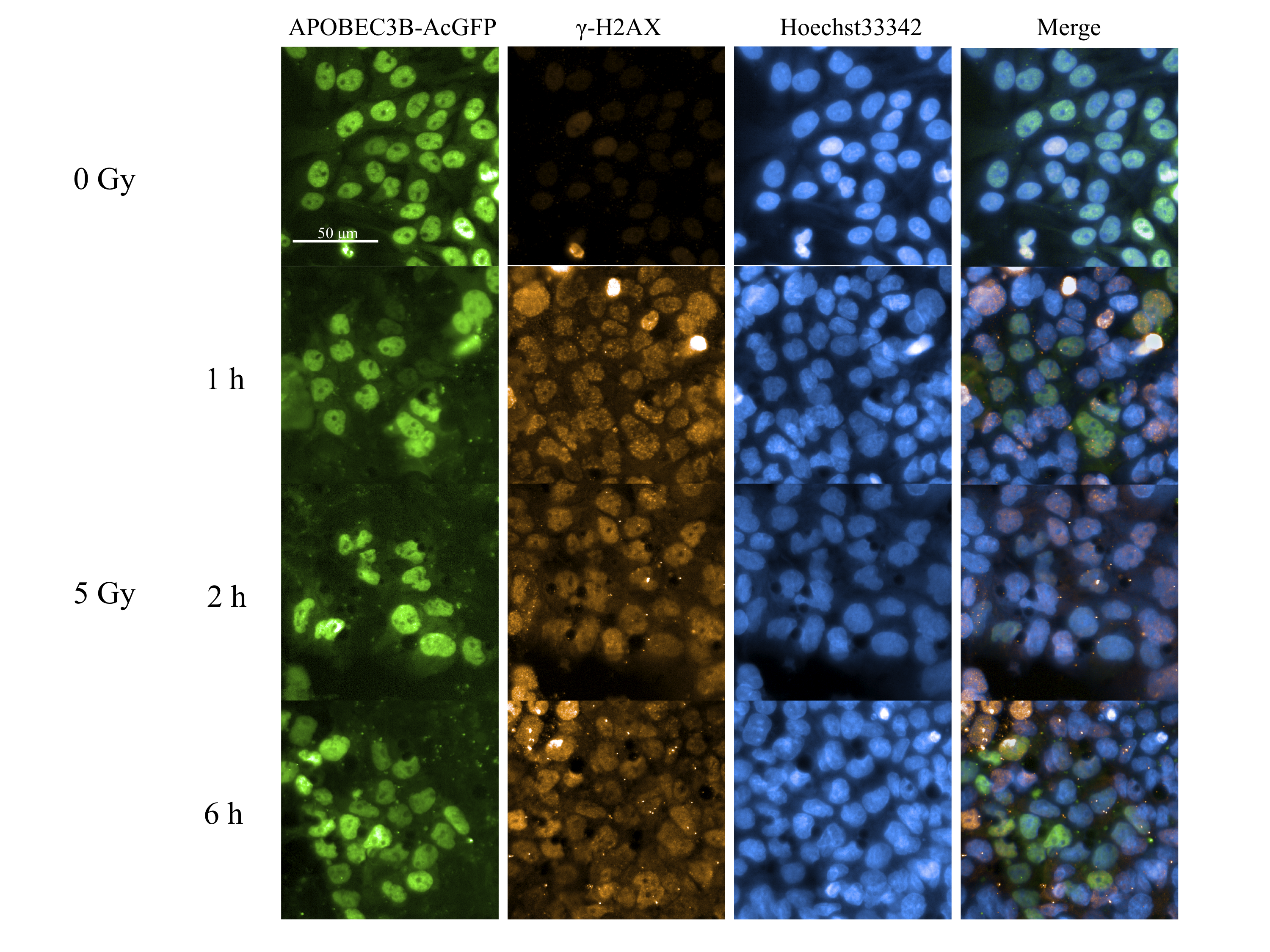

Supplement: S-Fig_7_rraa069 [file s-fig_7_rraa069.png]
